# Immunomonitoring of Tacrolimus in Healthy Volunteers: The First Step from PK- to PD-Based Therapeutic Drug Monitoring?

**DOI:** 10.3390/ijms20194710

**Published:** 2019-09-23

**Authors:** Aliede E. in ‘t Veld, Hendrika W. Grievink, Mahdi Saghari, Frederik E. Stuurman, Marieke L. de Kam, Aiko P. J. de Vries, Brenda C. M. de Winter, Jacobus Burggraaf, Adam F. Cohen, Matthijs Moerland

**Affiliations:** 1 Centre for Human Drug Research, 2333 CL, Leiden, The NetherlandsWGrievink@chdr.nl (H.W.G.); MSaghari@chdr.nl (M.S.); RStuurman@chdr.nl (F.E.S.); MdeKam@chdr.nl (M.L.d.K.); KB@chdr.nl (J.B.); AC@chdr.nl (A.F.C.); 2 Division of Nephrology, Department of Internal Medicine, Leiden University Medical Center, 2333 ZA, Leiden, The Netherlands; A.P.J.de_Vries@lumc.nl; 3 Department of Hospital Pharmacy, Erasmus Medical Center, 3014 GD, Rotterdam, The Netherlands; B.deWinter@erasmusmc.nl; 4 Division of Pharmacology, Leiden Academic Centre for Drug Research, 2333 CC, Leiden, The Netherlands; 5 Department of Surgery, Leiden University Medical Center, 2333 ZA, Leiden, The Netherlands

**Keywords:** immunosuppressive drugs, transplantation, pharmacodynamics, pharmacokinetics, tacrolimus, therapeutic drug monitoring, immunomonitoring

## Abstract

Therapeutic drug monitoring is routinely performed to maintain optimal tacrolimus concentrations in kidney transplant recipients. Nonetheless, toxicity and rejection still occur within an acceptable concentration-range. To have a better understanding of the relationship between tacrolimus dose, tacrolimus concentration, and its effect on the target cell, we developed functional immune tests for the quantification of the tacrolimus effect. Twelve healthy volunteers received a single dose of tacrolimus, after which intracellular and whole blood tacrolimus concentrations were measured and were related to T cell functionality. A significant correlation was found between tacrolimus concentrations in T cells and whole blood concentrations (r = 0.71, *p* = 0.009), while no correlation was found between tacrolimus concentrations in peripheral blood mononuclear cells (PBMCs) and whole blood (r = 0.35, *p* = 0.27). Phytohemagglutinin (PHA) induced the production of IL-2 and IFNγ, as well as the inhibition of CD71 and CD154 expression on T cells at 1.5 h post-dose, when maximum tacrolimus levels were observed. Moreover, the in vitro tacrolimus effect of the mentioned markers corresponded with the ex vivo effect after dosing. In conclusion, our results showed that intracellular tacrolimus concentrations mimic whole blood concentrations, and that PHA-induced cytokine production (IL-2 and IFNγ) and activation marker expression (CD71 and CD154) are suitable readout measures to measure the immunosuppressive effect of tacrolimus on the T cell.

## 1. Introduction

A combination of tacrolimus, mycophenolate mofetil (MMF), and glucocorticoids is the standard treatment of choice for kidney transplant recipients. However, despite the excellent survival rate (>90%) in the first year after transplantation in treated recipients, long-term clinical outcomes remain poor [1,2]. Calcineurin inhibitors (CNIs), like tacrolimus, suffer from large intra- and inter-patient variability in pharmacodynamic (PD) activity, complicating optimization of an individual dosing strategy [3]. Underexposure can lead to acute organ rejection and formation of donor-specific antibodies, while overexposure is associated with an increased risk of infection, toxicity, and malignancies.

The calcineurin inhibitor tacrolimus (FK506) is generally used after allogeneic organ transplantation, but also in other T cell-mediated diseases such as eczema and psoriasis. In order to maintain optimal levels of tacrolimus and to minimize the risk of overexposure, therapeutic drug monitoring (TDM) of pre-dose trough levels (C_0_) in whole blood is routinely performed in kidney transplant recipients. Nonetheless, toxicity and rejection still occur in patients within the C_0_-range, which indicates that the relationship between C_0_ measurements and the occurrence of rejection or tacrolimus-related toxicity is debatable [4,5]. Alternatively, the monitoring of intracellular drug concentrations in peripheral blood mononuclear cells (PBMCs) can be informative, although its correlation with clinical outcomes is suboptimal [6].

The mechanism of action of tacrolimus involves complex formation with the intracellular FK506 binding protein (FKBP12). This complex binds and inhibits calcineurin phosphatase activity, which causes a reduction in the expression of nuclear factor of activated T cells (NFAT)-mediated pro-inflammatory genes, such as interleukin 2 (IL-2) and interferon gamma (IFNγ) [7]. The quantitative relationship between tacrolimus concentration and the effect on T cell functionality has been extensively studied, also in primary human cells [8,9,10,11,12]. However, the effect of tacrolimus in fresh human whole blood samples has been left unattended, while the availability of a whole blood PD assay could be the missing link in TDM of tacrolimus. Unraveling the relationship between T cell functionality and tacrolimus dose, and tacrolimus concentration in whole blood and in the target cell would enable a PD- rather than a pharmacokinetic (PK)-based approach for future TDM of tacrolimus.

In the current study we aimed to investigate the relationship between tacrolimus dose, tacrolimus concentration, and its effect on the target cell. We developed functional immune tests for the quantification of tacrolimus effects in stimulated human whole blood samples. In an open label study, healthy volunteers received a single dose of tacrolimus, where after tacrolimus concentrations were measured in whole blood and isolated cells (PBMCs and T cells) and were related to T cell functionality (ex vivo cytokine production and flow cytometry-based cell activation). This study may be a first step towards the identification of functional PD readout measures for future immunomonitoring of transplantation patients, allowing adjustment of treatment regimens according to the needs of individual patients.

## 2. Results

### 2.1. Subject Characteristics

Twelve healthy volunteers, 6 men and 6 women, with a mean age of 31.5 years (range 18–54), participated in the study. Tacrolimus was well tolerated and there were no treatment-related adverse events.

### 2.2. Whole Blood and Intracellular Pharmacokinetics

**Figure 1:**
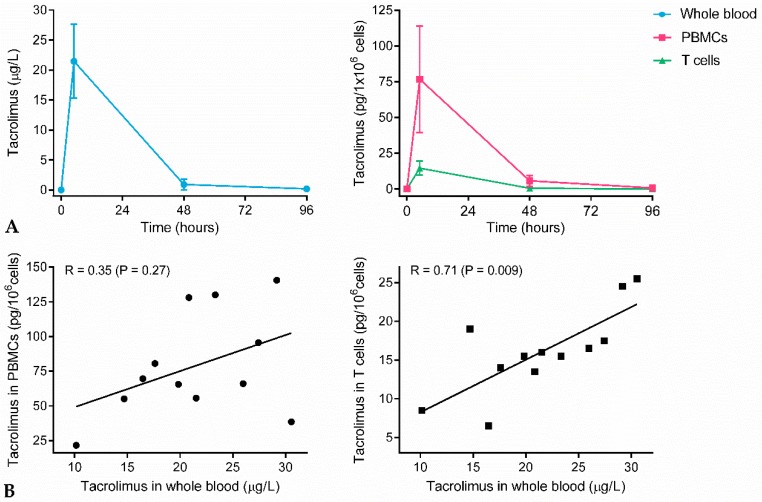
(**A**) Mean tacrolimus concentration over time in whole blood, PBMCs, and T cells. Samples were taken at 0 h, 1.5 h, 48 h, and 96 h post-dose. (**B**) Correlation between tacrolimus concentrations at 1.5 h post-dose in whole blood and PBMCs, and between whole blood and T cells.

PK profiles of tacrolimus in whole blood, PBMCs, and T cells are shown in Figure 1. In all matrices, the highest tacrolimus levels were observed at 1.5 h post-dose, and there was considerable variation between subjects (whole blood: 21.5 ± 6.2 μg/L, PBMCs: 76.8 ± 37.3 pg/10^6^ cells, T cells: 14.5 ± 4.9 pg/10^6^ cells). At 48 h after administration, tacrolimus concentrations almost returned to baseline levels. PK profiles were not significantly different in whole blood, PBMCs, and T cells. Intracellular tacrolimus concentrations, however, differed largely between T cells and PBMCs. The tacrolimus concentration in T cells was on average 5.3× lower compared to the concentration in PBMCs at 1.5 h post-dose.

Whole blood concentrations are generally used for therapeutic drug monitoring in renal transplantation patients. To determine whether whole blood levels may serve as proxy for drug concentrations that enter the target cell, whole blood concentrations were correlated to the intracellular concentrations. Tacrolimus levels in PBMCs showed no correlation with tacrolimus levels in whole blood, whereas tacrolimus levels in PBMCs were significantly correlated with tacrolimus levels in whole blood (r = 0.71, *p* = 0.009).

### 2.3. Cytokine Production

**Figure 2:**
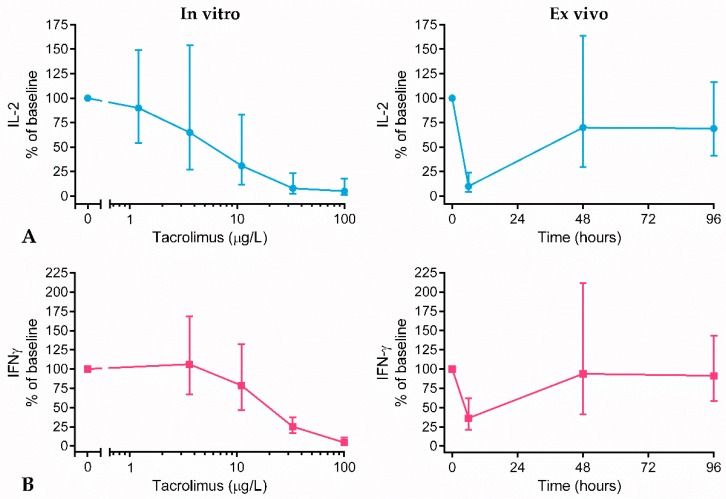
In vitro and ex vivo tacrolimus effect on cytokine production. (**A**) IL-2 production (blue lines) and (**B**) IFNγ production (red lines) in phytohemagglutinin (PHA)-stimulated whole blood. In vitro tacrolimus effect: pre-dose cytokine production after incubation with decreasing doses of tacrolimus (100, 33, 11, 3.7, and 1.2 μg/L). Ex vivo tacrolimus effect: cytokine production at 0 h, 1.5 h, 48 h, and 96 h after dosing. The cytokine production is calculated as percentage of baseline, and is displayed as mean ± SD.

To study the immunosuppressive effect of tacrolimus, cytokine production was measured after 24 h of phytohemagglutinin (PHA) stimulation in whole blood. PHA is a lectin known for its membrane glycoproteins binding, including the T cell receptor (TCR), which leads to the activation of T cells [13]. PHA stimulation was used to induce a general T cell response. Figure 2 shows the in vitro tacrolimus concentration–response curve that was generated pre-dose for each individual subject, and the ex vivo tacrolimus effect on cytokine production after dosing. A clear in vitro concentration–response relationship between tacrolimus and IL-2 and IFNγ production was found (IC50 of 5.6 µg/L and 18.6 µg/L, respectively), with a 95% inhibition of both cytokines at a tacrolimus concentration of 100 µg/L. Ex vivo, tacrolimus strongly inhibited cytokine production at 1.5 h post-dose (10.0% IL-2 and 36.3% IFNγ production remaining). The observed ex vivo cytokine inhibition corresponded well to the in vitro cytokine inhibition (Figure 2, left panels versus right panels, for tacrolimus concentration of 21.5 μg/L and 1.5 h time point).

**Figure 3:**
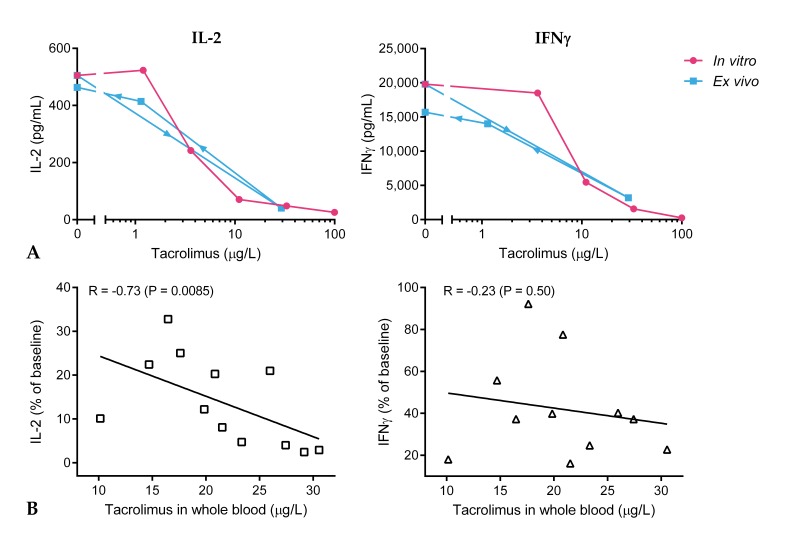
(**A**) Overlay of in vitro and ex vivo tacrolimus effect on cytokine production after 24 h whole blood stimulation with PHA for one subject. In vitro tacrolimus effect: pre-dose cytokine production after incubation with decreasing doses of tacrolimus (100, 33, 11, 3.7, and 1.2 μg/L). Ex vivo tacrolimus effect: cytokine production at 0 h, 1.5 h, 48 h, and 96 h after dosing. Arrows indicate time lapse. (**B**) Correlation between tacrolimus concentrations at 1.5 h post-dose in whole blood and relative IL-2 and IFNγ production.

Figure 3 shows the in vitro and ex vivo effect of tacrolimus in a single subject. At this individual subject level, the in vitro concentration–effect curve appears to be a good predictor of the ex vivo tacrolimus effect. Cytokine production and tacrolimus concentrations at 1.5 h were correlated, and relative IL-2 production was found to correlate significantly with whole blood tacrolimus concentration (Figure 3B, r = −0.73, *p* = 0.0085). T cell tacrolimus concentration also correlated with relative IL-2 production (r = –0.51, *p* = 0.09), while this was not confirmed for tacrolimus concentration in PBMCs (r = −0.03, *p* = 0.92). A stronger inhibition of IL-2 production was observed in subjects with increased tacrolimus levels at 1.5 h post-dose, as compared to subjects with decreased levels of tacrolimus. This correlation could not be confirmed for IFNγ production, which indicates that the inter-subject variability in IFNγ production at 1.5 h cannot be explained by the difference in tacrolimus concentration.

### 2.4. Surface Marker Expression

**Figure 4:**
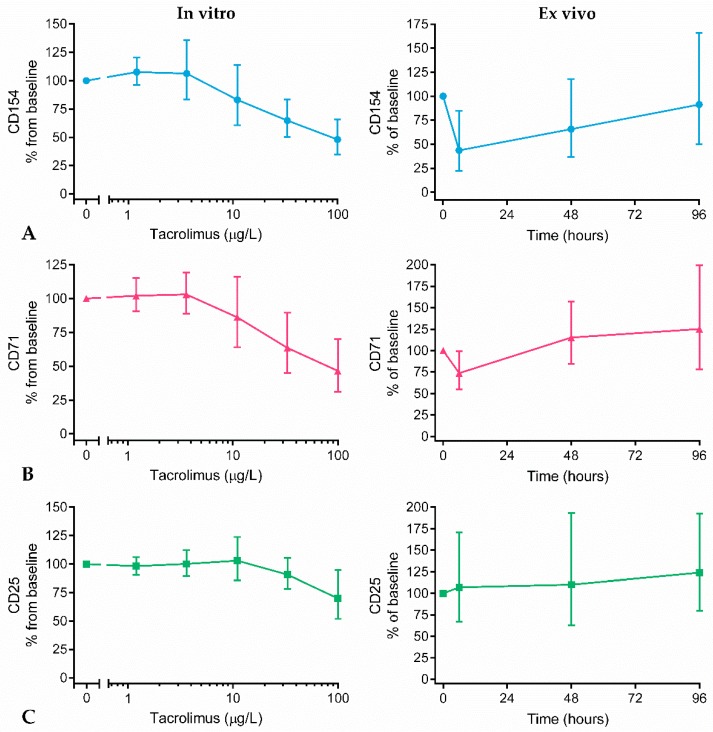
In vitro and ex vivo tacrolimus effect on expression of (**A**) CD154 (blue lines), (**B**) CD71 (red lines), and (**C**) CD25 (green lines) on CD3+ T cells after 48 h whole blood stimulation with PHA. In vitro tacrolimus effect: pre-dose surface marker expression after co-incubation with different tacrolimus concentrations (100, 33, 11, 3.7 and 1.2 μg/L). Ex vivo tacrolimus effect: surface marker expression over time, after PHA stimulation of whole blood samples collected from tacrolimus-exposed subjects. The expression was calculated as percentage of baseline, and is displayed as mean ± SD.

Tacrolimus concentration–effect curves were generated, showing the surface marker expression in pre-dose blood samples (in vitro drug effect), and in PHA-stimulated whole blood collected over time (ex vivo drug effect). Figure 4 shows that tacrolimus substantially and concentration dependently suppressed the expression of CD154 and CD71 in vitro. In contrast to PHA-induced cytokine release of IL-2 and IFNγ, tacrolimus did not fully inhibit PHA-driven surface marker expression of CD154 and CD71. Even at a concentration of 100 μg/L tacrolimus, a concentration that is never achieved in patients, a remaining surface marker expression of approximately 50% was found. Compared to the average expression found in unstimulated samples (a relative expression of 15% CD71, 17% CD154, and 24% CD25, compared to the stimulated sample), there was still surface marker expression remaining that could not be suppressed. The relative expression found in unstimulated samples was the background expression after 6 h incubation of the whole blood samples.

Of all measured activation markers, the expression of CD154 and CD71 on T cells was most strongly inhibited after dosing (44% and 73% remaining, respectively). The maximal ex vivo drug effect was observed at a whole blood concentration of 21.5 μg/L tacrolimus at 1.5 h post-dose, which corresponded with the observed in vitro effect size. CD25 expression, on the other hand, was not affected by tacrolimus dosing, which corresponded with the observed minor in vitro drug effect. A small in vitro tacrolimus effect on CD95 and CD69 expression was found, but CD95 and CD69 expressions were not significantly altered by ex vivo tacrolimus concentrations (Supplementary Figure S1).

No correlation was found between tacrolimus levels in whole blood and CD154 and CD71 expression at 1.5 h (Supplementary Figure S2).

### 2.5. Calcineurin Activity

Calcineurin phosphatase activity assessed, as described by Sellar et al. [14], allows for a PD readout measure more proximal to the drug target. However, this method proved to be unfeasible in the current study, mostly because sample handling was laborious and time-consuming. Data showed a high degree of variability and no dose–response relationship. The spectrophotometric readout of calcineurin activity was properly performed (CV of duplicates < 20%).

## 3. Discussion

Despite routinely performed therapeutic drug monitoring (TDM) in kidney transplant recipients, transplant rejection, infection, and (nephro)toxicity are still prevalent among patients with tacrolimus concentrations within the target range. Tacrolimus trough concentrations in whole blood are roughly targeted between 5 and 10 µg/L, based on a few randomized clinical trials showing correlations between target concentration and clinical outcome [15]. To improve the understanding of the relationship between tacrolimus concentration and the effect on clinical outcomes, new readout measures are of profound importance. We conducted a clinical study in healthy volunteers receiving a single dose of tacrolimus. Tacrolimus concentrations were quantified in whole blood, PBMCs, and T cells, and correlated with proximal drug effects (i.e., effects on the target cell).

At 1.5 h after drug administration, the highest tacrolimus concentrations were detected in whole blood, PBMCs, and T cells. At the next time point, 48 h after administration, tacrolimus concentrations had almost returned to baseline levels in all three matrices. This is in line with the reported PK profile of tacrolimus in healthy volunteers [16]. Our data indicate that tacrolimus did not stay significantly longer in target cells than freely circulating in blood. The intracellular tacrolimus concentrations were significantly different in PBMCs and T cells, even though it is known that the majority of PBMCs consist of T cells (60%) [17]. Presumably, the difference between tacrolimus concentrations in PBMCs and T cells were not caused by the isolation procedure but might have been caused by another PBMC subpopulation with significant tacrolimus uptake. Washing steps during PBMC isolation are known to diminish the effect of tacrolimus [18], but the number of washing steps were kept similar for both isolations. Since intracellular tacrolimus concentrations have never been measured in cell populations other than PBMCs, the identity of the other cell subpopulation remains unknown. There was no correlation between tacrolimus concentrations in whole blood and PBMCs at 1.5 h post-dose. Evidence supporting this finding is scarce and conflicting; some papers do report a correlation in transplantation patients [19,20], whereas other papers do not [21,22]. Tacrolimus levels in T cells, on the other hand, did correlate significantly with whole blood concentrations. This finding supports the chosen whole blood-based TDM strategy and indicates that tacrolimus concentration in whole blood is a good representation of the concentration in the target cell.

Clinical practice nonetheless showed that whole blood tacrolimus concentration is far from ideal as a primary measure of TDM. We hypothesized that drug activity rather than drug concentration may be more appropriate for future TDM. We took a first step towards this future perspective by selecting, optimizing, and qualifying functional assays for the quantification of the tacrolimus effect on the T cell. The results of the current study showed that the production of PHA-induced IL-2 and IFNγ, and the expression of CD71 and CD154 on T cells were the most promising pharmacodynamic readout measures for the quantification of the in vitro and ex vivo tacrolimus effect. PHA-induced cytokine release was almost completely inhibited at an in vitro concentration of 100 µg/L tacrolimus. At an in vitro concentration of 20–25 µg/L, which is the peak tacrolimus whole blood concentration in healthy volunteers, the estimated inhibition of cytokine production was 80% for IL-2 production and 50% for IFNγ production (25% and 50% cytokine release remaining, respectively). At this peak concentration, 1.5 h after administration, tacrolimus inhibited IL-2 production by 90% and IFNγ production by 64% ex vivo (10% and 36% cytokine release remaining, respectively). Though PK and PD data were not formally modeled and integrated (due to the limited sample size), this indicates that the in vitro tacrolimus effect on cytokine production corresponds decently with the ex vivo tacrolimus effect.

Tacrolimus significantly reduced the PHA-induced expression of CD71 and CD154 on T cells. For surface marker CD25, the tacrolimus effect was less obvious. Transferrin receptor (CD71), co-stimulatory molecule CD40 ligand (CD154), and IL-2 receptor (CD25) are all upregulated upon T cell activation [23], and their expression on lymphocytes has been associated with clinical outcomes in transplant recipients [24,25,26]. Our data showed that all surface markers were expressed by non-stimulated T cells (expression levels of 15%, 17%, and 24%, for CD71, CD154, and CD25, respectively, compared to a PHA-stimulated condition, set as 100%). At a concentration of 100 µg/L, tacrolimus reduced CD71 and CD154 expression to approximately 50% in vitro, which means that at this very high tacrolimus concentration, T cell activation was still not fully inhibited. The ex vivo tacrolimus effect on the expression of CD71 and CD154 in drug-exposed volunteers was 27% and 56% for CD71 and CD154 expression, respectively (73% and 44% remaining). The in vitro and ex vivo tacrolimus effect on activation marker expression corresponded well, after having compared the drug effect at 1.5 h (ex vivo; tacrolimus peak) with the drug effect at a tacrolimus concentration of 20–25 µg/L (in vitro).

Due to logistical reasons, the number of data points with significant tacrolimus concentrations and substantial T cell inhibition were limited, which hampered an analysis involving quantitative correlations or systematic PK/PD integration. However, the ex vivo tacrolimus effects in drug-exposed volunteers corresponded well with the in vitro tacrolimus effects quantified in baseline samples from the same volunteers. Since the in vitro concentration–effect curve seemed to predict the ex vivo tacrolimus effect in the current study, it might be an option to base future TDM in transplant recipients on the in vitro concentration–effect curve. A critical next step will be to investigate the correlation between the functional T cell measures and clinical outcomes, such as allograft survival/rejection and side effects, in a patient-based study.

Despite the explorative character of this study, it is tempting to speculate about the theoretical T cell activity profile over time in tacrolimus-treated transplantation patients, based on the current study outcomes. It is known that the PK profile of tacrolimus is highly variable between transplant recipients. Trough concentrations are measured 12 h after dosing and can vary between 0.6 and 50 µg/L, with an average of 5–10 µg/L [27,28,29,30]. Based on the in vitro tacrolimus effect we observed in the current study (which corresponded well with the ex vivo drug effect after treatment of healthy volunteers), such concentrations would translate into a minor inhibition of T cell activity: at a tacrolimus whole blood concentration of 5 µg/L, even none of the PD T cell measures were inhibited, except for IL-2 production (approximately 40% inhibition). These findings suggest that with the conventional tacrolimus dosing regimen, some patients may experience time intervals in which their T cell activity is not inhibited (at least not by tacrolimus). On the other hand, in this study the effect of a single dose of tacrolimus was studied, while in patients the responsiveness of T cells might be different because of long-term repeated tacrolimus dosing. Moreover, tacrolimus is usually combined with MMF and corticosteroid treatment, drugs that also suppress T cell activity, but that were not included in the current study. For these drugs, and combinations of these drugs, a dedicated PK/PD study in healthy volunteers should be performed.

In conclusion, this study showed that intracellular tacrolimus concentrations mimic the time course of whole blood concentrations, and that PHA-induced cytokine production (IL-2 and IFNγ) and activation marker expression (CD71 and CD154) are suitable PD readout measures for the quantification of the immunosuppressive effect of tacrolimus on the T cell. Although the effect of tacrolimus on T cell activity has been studied before [8,9,10,11], this is the first study in which whole blood and intracellular tacrolimus concentrations are related to ex vivo drug effects, and in which in vitro and ex vivo tacrolimus effects are compared. As such, the current study may serve as the first step from PK- towards PD-based therapeutic drug monitoring in transplant recipients using tacrolimus.

## 4. Materials and Methods

### 4.1. Study Design

In this open label study, 12 healthy volunteers received a single oral dose of 0.05 mg/kg Prograf^®^, rounded up to the available dosage forms (0.5 mg, 1 mg, and 5 mg Prograf^®^). The dosage was based on the recommended dose for renal transplant patients receiving both tacrolimus and mycophenolate mofetil treatment. The healthy volunteers were both male and female, between 18 and 55 years of age and non-smoking. All subjects gave written informed consent and did not have any disease associated with immune system impairment or evidence of any other active or chronic disease. Volunteers were excluded when taken any other drugs within 21 days prior to study start. This study was approved by the independent medical ethics committee “Medisch Ethische Toetsingscommissie van de Stichting Beoordeling Ethiek Biomedisch Onderzoek” (Assen, the Netherlands), and is registered with the Dutch Trial Registry (Nederlands Trial Register) under study number NTR7420.

### 4.2. Whole Blood and Intracellular PK

Blood samples were drawn pre-dose and 1.5, 48, 96, and 192 h after drug administration. The samples for whole blood PK measurement were collected in K2EDTA tubes (Becton Dickinson, Franklin Lakes, NJ, US) and stored at −80 °C. PBMCs were collected using sodium heparin CPT tubes (Becton Dickinson), and T cells were isolated from heparinized whole blood by immunomagnetic cell sorting. The RoboSep human T cell isolation kit was used in combination with RoboSep (StemCell Technologies) to label unwanted cells with antibody complexes and magnetic particles, after which T cells were isolated by automated magnetic sorting. After PBMC and T cell isolation, the cells were washed, and the remaining red blood cells were removed using RBC lysis buffer (Thermo Fisher Scientific, Waltham, MA, US). PBMCs and T cells were counted with a MacsQuant 10 analyzer (Miltenyi Biotec, Bergisch Gladbach, Germany) and stored in PBS at 20 × 10^6^ cells/mL at −80 °C. The purity of the isolated T cell population was assessed with an anti-CD45-FITC and anti-CD3-VioGreen staining (Miltenyi Biotec).

Whole blood and intracellular tacrolimus concentrations were measured using a Waters Acquity UPLC–MS/MS system by the Department of Hospital Pharmacy, Erasmus Medical Center, as described previously [19].

### 4.3. In Vitro and Ex Vivo Whole Blood Culture

On the same time points as the PK samples, blood was drawn for PD assessments, including whole blood challenges. All incubations were started within one hour after blood withdrawal. For the measurement of cytokine production and surface marker expression, heparinized whole blood (Becton Dickinson) was stimulated with 10 μg/mL phytohemagglutinin (PHA) (Sigma Aldrich, St. Louis, MO, US). To generate an in vitro tacrolimus concentration–effect curve for every individual subject, pre-dose samples were incubated with PHA and concentrations of 100, 33, 11, 3.7, and 1.2 μg/L tacrolimus (Prograf^®^ for injection). To study the immunosuppressive effect of tacrolimus ex vivo, whole blood samples were incubated with PHA only.

Samples for the measurement of calcineurin activity were collected in EDTA tubes (Becton Dickinson). Pre-dose samples were first incubated 1 h at 37 °C, 5% CO_2_ with a concentration range of tacrolimus. For post-dose samples, the analysis was started directly (within one hour) after the blood sample was taken.

### 4.4. Cytokine Production

Whole blood samples were cultured for 24 h, after which supernatant was collected and stored at −80 °C until analysis. IFNγ and IL-2 concentrations were measured by the Meso Scale Discovery Vplex-2 method by Ardena Bioanalytical Laboratory in Assen, the Netherlands.

### 4.5. Surface Marker Expression

**Figure A1:**
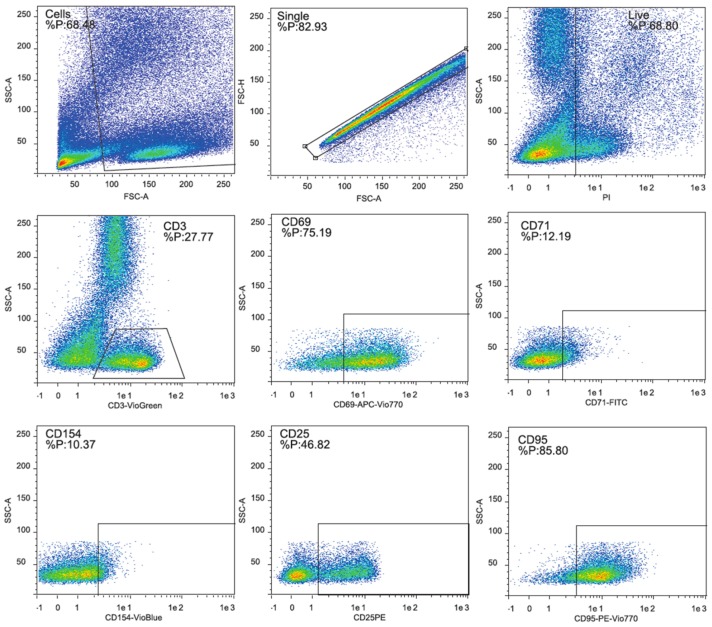
Gating strategy for surface marker expression on CD3+ T cells.

Following 48 h, whole blood incubation, RBC lysis buffer (Thermo Fisher Scientific) was used to lyse the red blood cells. After washing with PBS, cells were stained with anti-CD3-Viogreen, anti-CD69-APCVio770, anti-CD95-PEVio770, anti-CD25-PE, anti-CD71-FITC, and anti-CD154-VioBlue (Miltenyi Biotec). The samples were measured after a final washing step, using a MaqsQuant10 analyzer. Before measurement, propidium iodide (PI) (Miltenyi Biotec) was added to assess viability. Analysis of the cell populations was performed with Flowlogic software (Inivai Technologies, Mentone VIC, Australia). For each time point the unstimulated samples were used to set the correct gating. The gating strategy is shown in Figure A1 in Appendix A.

### 4.6. Calcineurin Activity

The method described by Sellar et al. was used for the measurement of calcineurin activity [14]. Red blood cells were lysed using ACK lysis buffer (Thermo Fisher Scientific, Waltham, MA, USA), after which two million cells were lysed in freshly prepared lysis buffer (50 mM Tris-HCL, 1.0 mM 1,4-Dithiothreitol, 5.0 mM L-ascorbic acid, 0.02% Igepal CA-630, 50 mg/L soybean trypsin inhibitor, 50 mg/L phenylmethylsulfonyl fluoride, 5.0 mg/L leupeptin, and 5.0 mg/L aprotinin). After lysis, the samples underwent three freeze–thaw cycles and were stored at −80 °C until use.

A calcineurin phosphatase activity kit (Enzo Life Sciences, Brussels, Belgium) was used to measure calcineurin activity according to the protocol described by Sellar et al. [14]. Phosphatase activity of calcineurin (pmol min^−1^ × 10^6^ cells) was calculated by subtracting the phosphate activity that was measured in the presence of Ca^2+^ and calmodulin from the phosphatase activity measured in the presence of EGTA.

### 4.7. Data Analysis

Data are presented as mean value ± standard deviation (SD). Correlations between tacrolimus concentrations, surface marker expression, and cytokine production were analyzed by Spearman′s rank-order correlation with SAS 9.4 (SAS Institute Inc., Cary, NC, USA). IC50 of in vitro cytokine production and surface marker expression was calculated using GraphPad Prism 6.05 (GraphPad software Inc., San Diego, CA, US).

## Supplementary Materials

Supplementary materials can be found at https://www.mdpi.com/1422-0067/20/19/4710/s1.

## Abbreviations

CNI	Calcineurin inhibitor
C_0_	Pre-dose trough concentration
FK506	Tacrolimus
FKBP	FK506 binding protein
IC50	Half maximal inhibitory concentration
IFNγ	Interferon gamma
IL-2	Interleukin 2
MMF	Mycophenolate mofetil
NFAT	Nuclear factor of activated T cells
PBMCs	Peripheral blood mononuclear cells
PD	Pharmacodynamics
PHA	Phytohemagglutinin
PI	Propidium iodide
PK	Pharmacokinetics
SD	Standard deviation
TDM	Therapeutic drug monitoring
WB	Whole blood
