# Supplementary material for: Immunomonitoring of Tacrolimus in Healthy Volunteers: The First Step from PK- to PD-Based Therapeutic Drug Monitoring?
Source: Int J Mol Sci. 2019 Sep 23;20(19):4710. doi: 10.3390/ijms20194710 (PMC6801784; doi:10.3390/ijms20194710)

T cell activation: CD69 expression in vitro (top) and ex vivo (bottom)

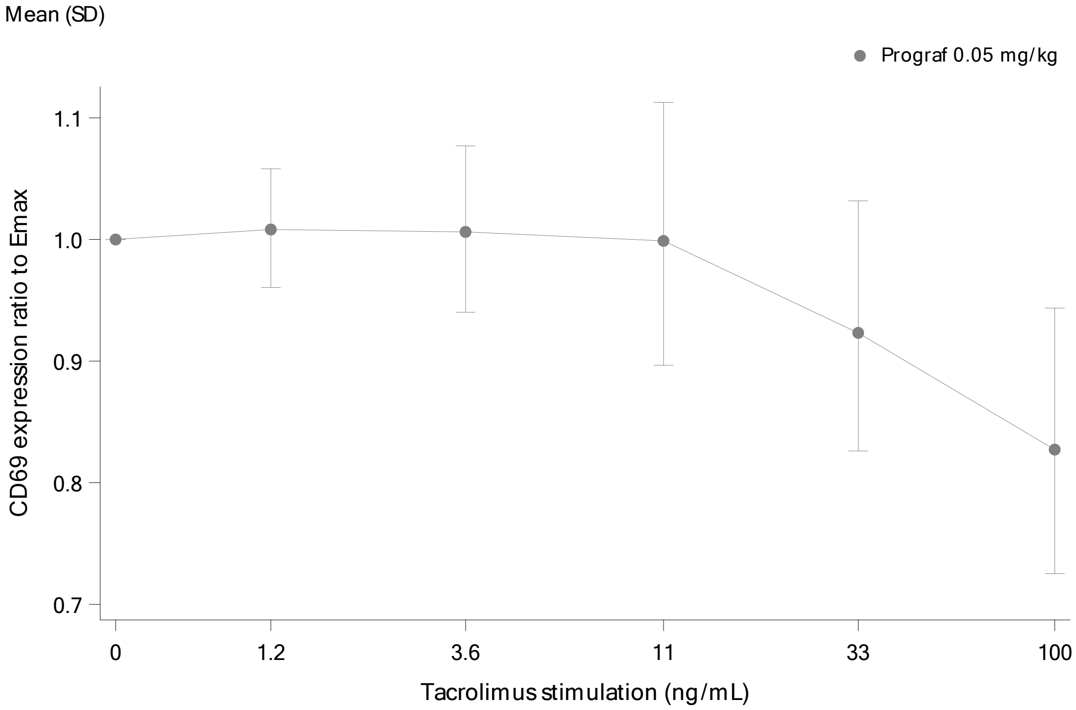

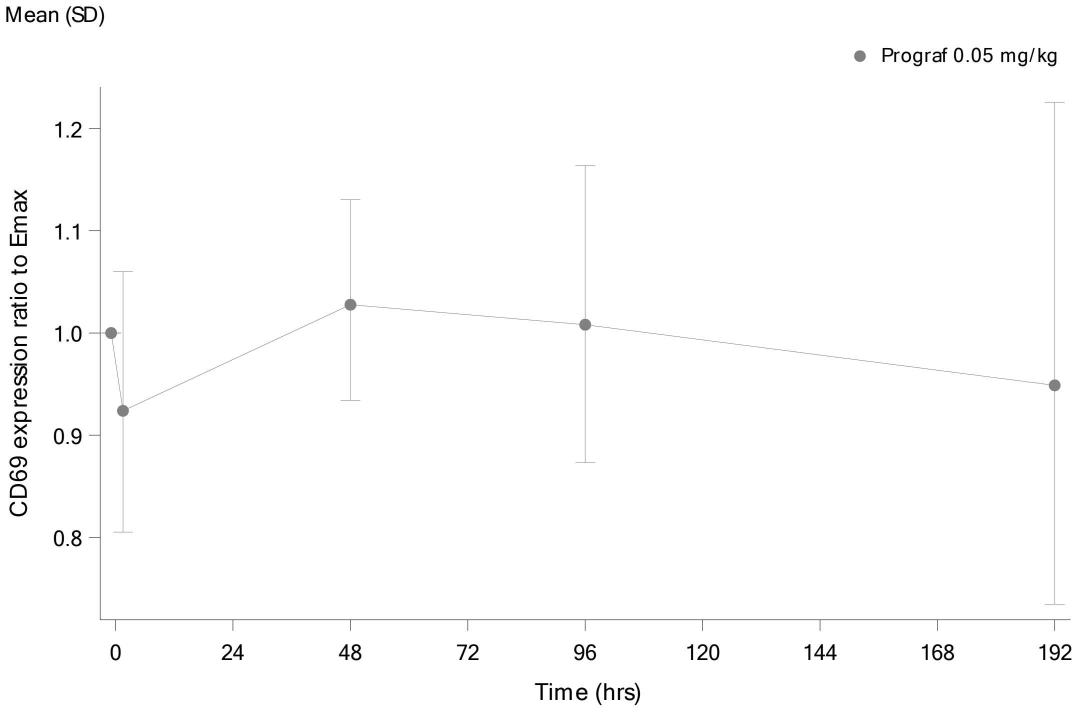

T cell activation: CD95 expression in vitro (top) and ex vivo (bottom)

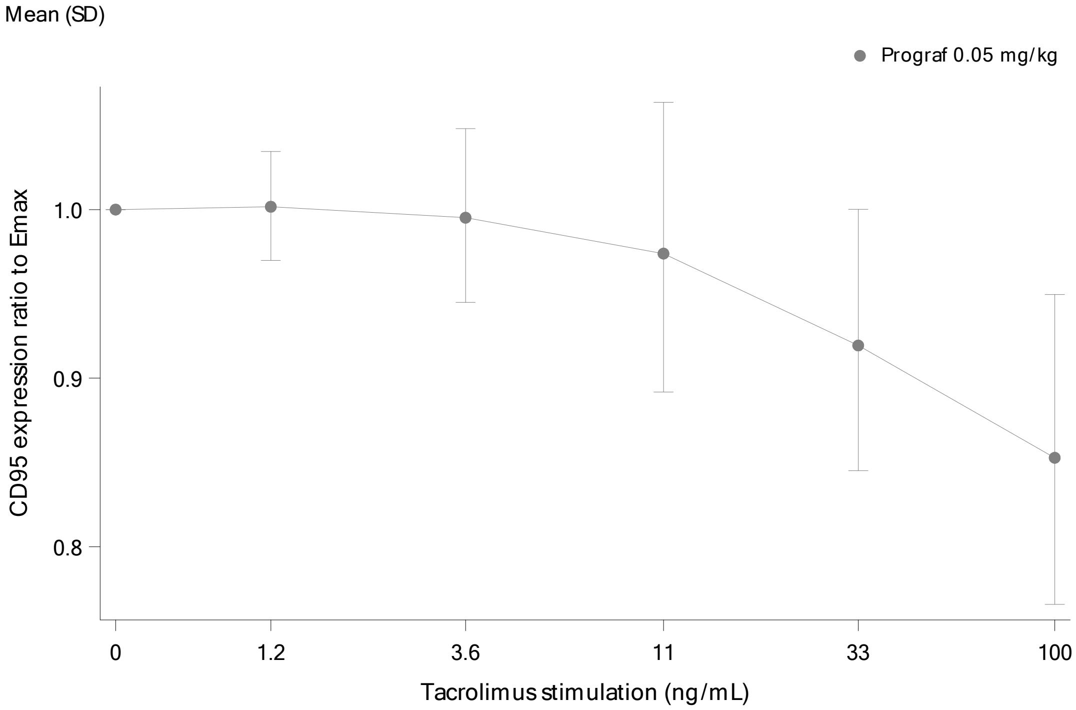

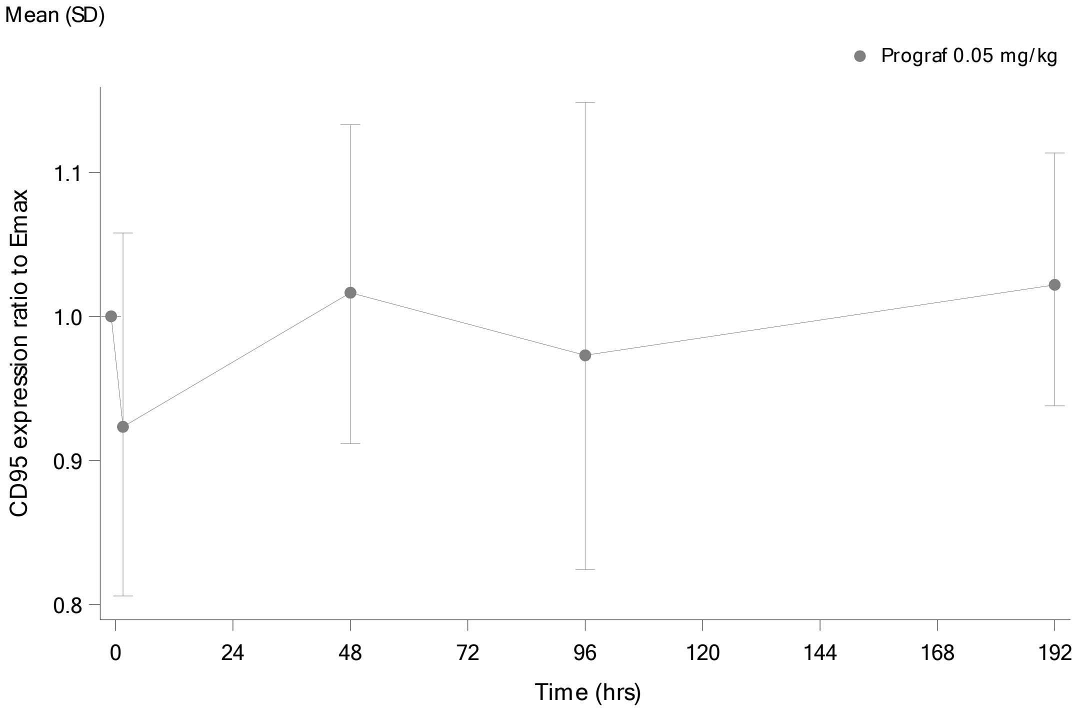

Tacrolimus concentration in whole blood versus CD71 (top) and CD154 (bottom) expression

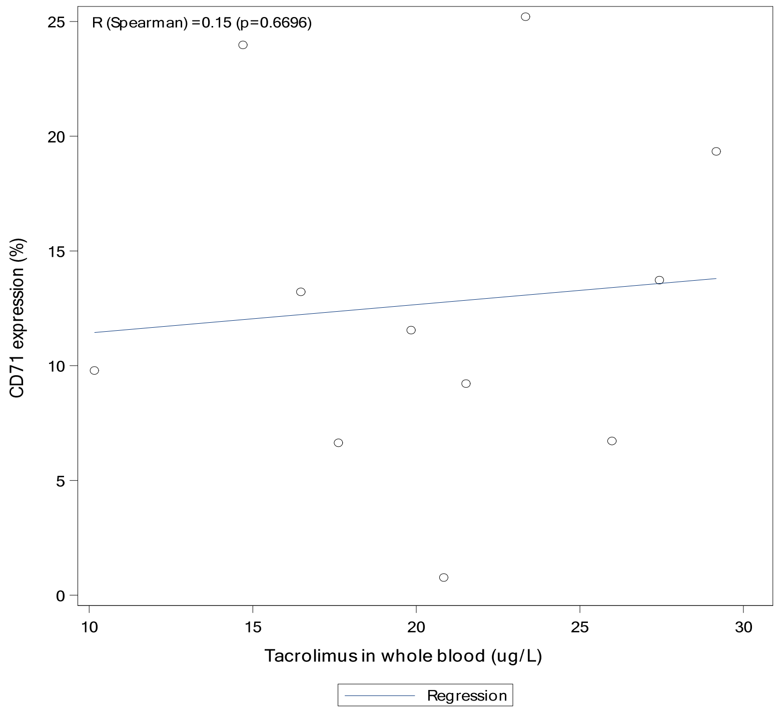

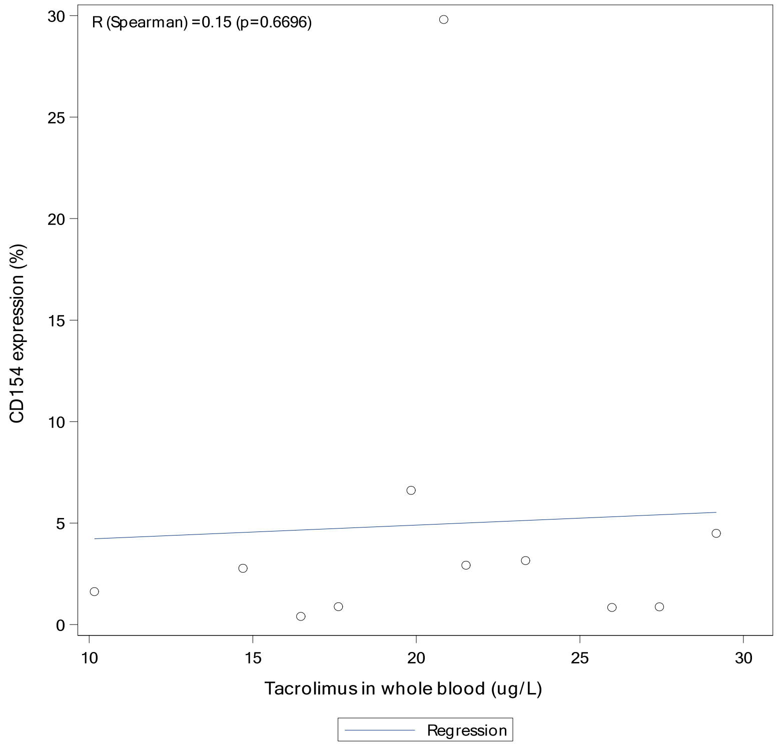

Supplement: Supplementary file 1 [file ijms-20-04710-s001.pdf]
